# Triptolide as a novel agent in pancreatic cancer: the validation using patient derived pancreatic tumor cell line

**DOI:** 10.1186/s12885-018-4995-0

**Published:** 2018-11-12

**Authors:** Seung Tae Kim, Sun Young Kim, Jeeyun Lee, Kyung Kim, Se Hoon Park, Young Suk Park, Ho Yeong Lim, Won Ki Kang, Joon Oh Park

**Affiliations:** Division of Hematology-Oncology, Department of Medicine, Samsung Medical Center, Sungkyunkwan University School of Medicine, 81 Irwon-ro, Gangnam-gu, Seoul, 06351 South Korea

**Keywords:** Patient derived cells (PDCs), Triptolide, Chk2

## Abstract

**Background:**

Triptolide induces apoptosis and DNA damage followed by inhibition of DNA repair associated gene expression. However, there is the limited data for biomarker to predict the benefit to triptolide in various cancers including pancreatic cancer.

**Methods:**

We investigated the anti tumor efficacy of triptolide in various pancreatic cancer cell lines (Capan-1, Capan-2, SNU-213, SNU-410, HPAFII, and Hs766T) and patient derived cells (PDCs) from metastatic pancreatic cancer patients.

**Results:**

In vitro cell viability assay for triptolide in 6 PC cell lines, the IC_50_ was 0.01 uM, 0.02 uM, 0.0096 uM for triptolide in Capan-1, Capan-2 and SNU-213. However, the growth of tumor cells was not significantly reduced by triptolide in Hs766T, SNU-410 and HPAFII. The distinct difference of gene expression was also observed between Capan-1, Capan-2 and SNU-213 and Hs766T, SNU-410 and HPAFII. In analysis of pathway using gene expression profiles, the integrin mediated RAS signaling pathway was associated with the sensitivity of the triptolide in PC cell lines. Immunoblot assay showed that Chk2 phosphorylation after triptolide was distinctively observed in SNU-213 sensitive to triptolide but, not in SNU-410 insensitive to triptolide. This finding in immunoblot assay was also reproduced in PDCs originated from pancreatic cancer patients.

**Conclusions:**

Our findings might be helpful to completely capture the subset of patients who may benefit to tripolide (minnelide). More robust biomarkers such as KRAS mutation and Chk2 phosphorylation and careful clinical trial design using triptolide (minnelide) are warranted.

**Electronic supplementary material:**

The online version of this article (10.1186/s12885-018-4995-0) contains supplementary material, which is available to authorized users.

## Introduction

Pancreatic cancer is an aggressive and highly fatal malignancy affecting a large number of individual worldwide, and survival 5 years after diagnosis is less than 5% with only 15% for the patients eligible for surgical resection at presentation [1–3]. Various factors cause this poor prognosis, including aggressive tumor biology and drug resistance to pancreatic tumor cells [4–6]. Although some chemotherapeutic agents such as gemcitabine, erlotinib, abraxene, oxaliplatin and irinotecan have taken to survival advantage, there is still urgent needs to discover and develop more effective treatment-options against pancreatic cancer [7, 8].

KRAS mutation has been known as being present in 70~ 95% of pancreatic cancers [9]. KRAS mutation has been known as crucial marker for growth and maintenance of pancreatic cancers and targeting the KRAS is inevitable component for realizing precision medicine to pancreatic cancers. Tripolide is a diterpenoid triepoxide derived from the herb Tripterygium wilfordii, which has been utilized as a natural agent [10]. Triptolide has shown great promise in preclinical studies using immotalized pancreatic cancer lines in immunocompromised mouse model [11]. Triptolide induces apoptosis and DNA damage followed by inhibition of DNA repair associated gene expression [12–14]. However, there is the limited data for biomarker to predict the benefit to triptolide in various cancers including pancreatic cancer.

Herein, we investigated the anti tumor efficacy of triptolide in various pancreatic cancer cell line. Additionally, we also intended to analyze the difference of signal pathway based on gene expression and conduct the immunoblot assay according to the status of sensitivity to triptolide. Further, anti-tumor effect of triptolide was verified through PDCs from metastatic pancreatic cancer patients with and without KRAS mutation.

## Material and methods

### Patient

This investigation was conducted in accordance with the ethical standards of the Declaration of Helsinki and national and international guidelines, and was approved by the Institutional Review Board at Samsung Medical Center. Between October 2013 and Jan 2016, patients with gastrointestinal cancer, rare cancer, and lung cancer prospectively enrolled in the SMC Oncology Biomarker study (Ref. No. 2011-07-089). The PDC culture protocol was performed for eligible patients. All of the primary tumors from the patients who provided written informed consent were genomically sequenced. In parallel, PDC establishment was attempted for all sequenced tumors.

### Cell lines and patient-derived cell culture

Triptolide was treated on six pancreatic cancer cell lines in vitro. Lines Hs766T and HPAFII were obtained from ATCC (American Type Culture Collection, Rockville, MD, USA). Lines SNU-410, Capan-1, Capan-2, and SNU-213 were obtained from KCLB (Korean Cell Line Bank, Seoul, Korea). Cells were cultured in RPMI, DMEM, or MEM media according to the manufacturer’s methods, supplemented with 10% of fetal bovine serum and 1% of antibiotic-antimycotic solution (Gibco, Carlsbad, CA, USA).

For culture of patient derived pancreatic cancer cells, malignant effusions or tumor tissue biopsy from patients with metastatic cancer was collected from patients who had provided informed consent. Collected effusions (1–5 L) were divided into 50 mL tubes, centrifuged at 1500 rpm for 10 min, and washed twice with PBS. Cell pellets were resuspended in culture medium and plated into 75 cm2 culture flasks. Collected tissue was minced and dissociated with enzymatic method. The cells were grown in RPMI 1640 supplemented with 10% fetal bovine serum and 1% antibiotic-anti-mycotic solution, 0.5 μg/ ml of hydrocortisone (Sigma Aldrich, St. Louis, MO, USA), 5 μg/ ml of insulin (PeproTech, Rocky Hill, NJ, USA), 5 ng of EGF and 2.5 ng of FGF (PeproTech). The medium was changed every 3 days, and cells were maintained at 37 °C in a humidified 5% CO_2_ incubator. PDCs were detached using TrypLE Express (Gibco BRL) to subculture when the cells reached 80–90% confluence.

### Cell treatment and viability assay

**Fig. 1:**
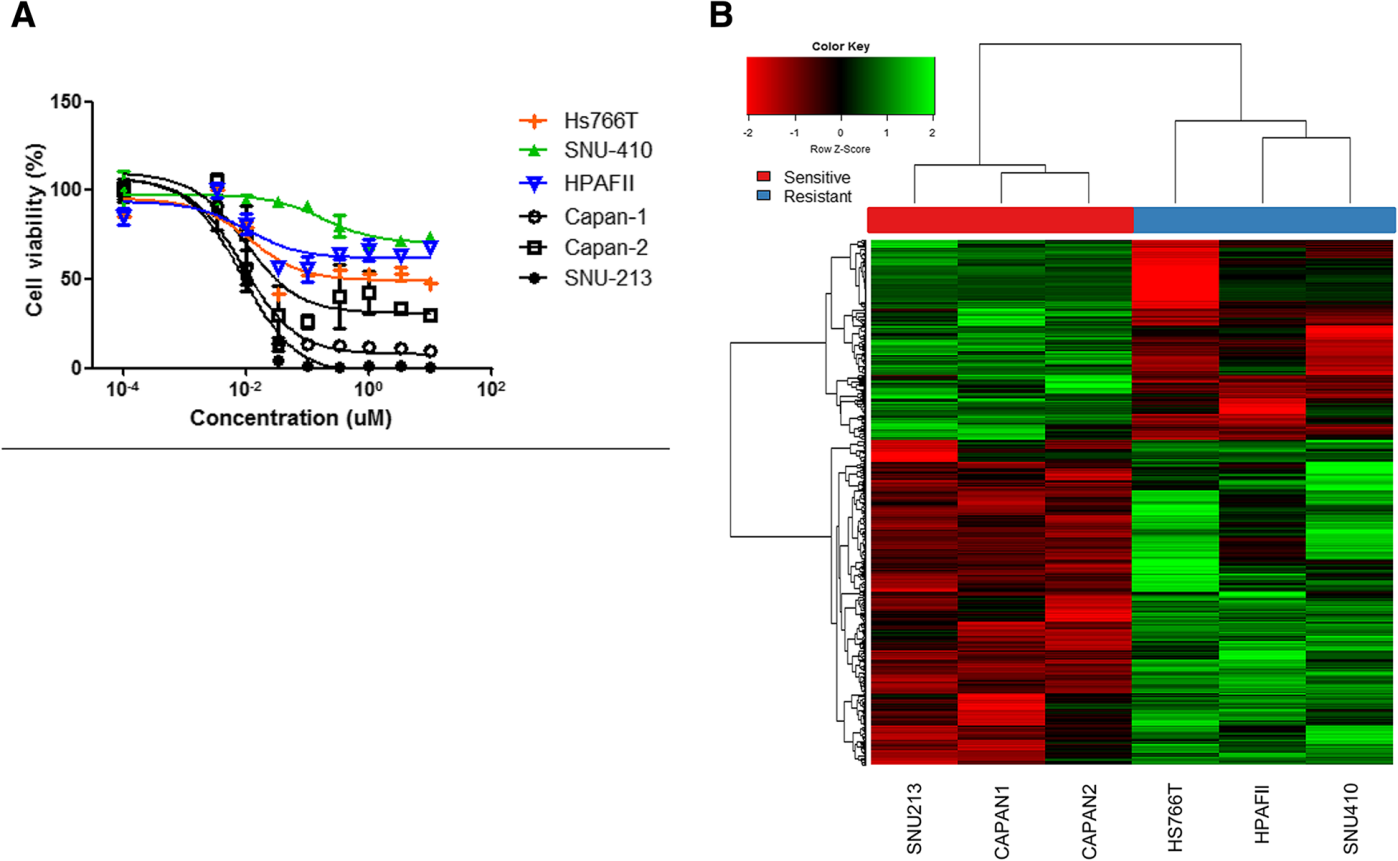
**a** Cell proliferation inhibition curve of triptolide on pancreatic cell lines. **b** A heat map generated using the 529 DEGs between triptolide sensitive cell line and resistant cell line. A heat map was constructed using the genes based on Pearson correlation distance and average linkage method

To observe the effect of triptolide, cells were seeded on 1~ 2 × 10^6^ cells/10 mm dishes or 5000 cells/ well/ 96-well plate for analysis of immunoblotting and cell proliferation inhibition assay. Cells were treated for 3~ 5 days with various doses of drugs as indicated in the Fig. 1. Cell proliferation inhibition was determined via Cell Titer Glo (Promega, Madison, WI, USA) according to the manufacturer’s protocol.

### Affymetrix microarray analysis

The array data for GSE36133, were downloaded from the Gene Expression Omnibus (GEO; http://www.ncbi.nlm.nih.gov/geo/) database, as reported by CCLE [15]. A total of 917 samples were used in the development of the Affymetrix microarray data. The expression profiles analyzed in this work were derived from six samples, including three samples of triptolide sensitive cell line and three samples of three samples of triptolide resistant cell line. The raw CEL data and annotation files were downloaded based on the GPL15308 platform for further analysis.

### Data processing and DEG analysis

The raw expression data were preprocessed using the gcrma algorithm with application of the EMA package in the R statistical software (version 3.1.2; Bell Labs, Murray Hill, NJ, USA) [16]. When multiple probes corresponded to the same gene, the mean value was calculated as the expression value of that gene.

The DEGs between triptolide sensitive cell line and resistant cell line were analyzed using the runWilcox. |log of fold change| > 1 were considered to be the cut-off values for DEGs screening. The 529 DEGs were ranked based on their Wilcoxon *P*-values and the markers with the lowest *P*-values were selected.

### Gene ontology (GO) and pathway enrichment analysis

The gene sets enriched in specific subtypes were further applied to ClueGO tool in Cytoscape to visualize significant gene ontology hierarchy and functional interaction maps between DEGs [17, 18].

The DEGs were classified into three GO categories, including molecular function (MF), biological process (BP) and cellular component (CC). *P* < 0.05 was set as the threshold value.

### Immunoblot analysis

Total proteins from cells were isolated by cOmplete Lysis-M (Roche) containing a protease inhibitor cocktail (Roche, Mannheim, Germany) and phosphatase inhibitor cocktail (Roche), and protein concentrations were determined according to Bradford procedure using a Quick Start Bradford Protein Assay (Bio-Rad, Hercules, CA, USA). Thirty micrograms of proteins were subjected to 10% SDS-polyacrylamide gel electrophoresis, and electro-transferred onto nitrocellulose membranes. The membranes were blocked with 5% nonfat dry milk in Tris-buffered saline containing 0.1% *v*/v Tween 20, and probed overnight at 4 °C with a Specific antibodies: phospho-ATM (Ser1981), ATM (D2E2), phospho-ATR (Ser428), ATR (E1S3S), phospho-Chk1 (Ser345), Chk1 (2G1D5), phospho-Chk2 (Thr68), Chk2 (1C12), Cyclin A2 (BF683), Cyclin D1 (92G2), and Cyclin E2 from Cell Signaling Technology (Beverly, MA, USA), Integrin beta1 from abcam (Cambridge, UK) and beta actin from Sigma Aldrich. Horseradish peroxidase-conjugated anti-rabbit or mouse IgG (Vector, Burlingame, CA, USA) were used as a secondary antibody, and signals were detected by chemiluminescence using ECL Western Blotting Substrate (Thermo Scientific, Rockford, IL, USA), and visualized by using LAS-4000 (Fujifilm, Tokyo, Japan).

### Droplet digital PCR test of KRAS mutations

KRAS G12 V mutation levels of patient derived cells were measured using a ddPCR system (Bio-Rad Laboratories, Hercules, CA, USA). Briefly, sequence of primers and probes for detection were provided from Taq man (assay ID: KRAS_520). Total 20 ul of PCR mixture was loaded into a 24-well consumable droplet generation cartridge. The water-in-oil emulsions were transferred to a 96-well PCR plate and subjected to amplification cycles. (Cycling protocol: 95 °C for 10 min, 40 cycles of 94 °C for 30 s and 60 °C for 1 min, and a final step at 98 °C for 10 min). Then plates were loaded into the com-mercially obtained QX100 droplet reader (Bio-Rad). Concentrations of targets in the samples were analyzed by using QuantaSoft software as manufacturer’s instruction.

### Statistical method

For cell viability curves, results are expressed as the means. Paired one way ANOVA tests were used to calculate the *P* values. Statistical significance was assessed using one way ANOVA tests and described in the figure.

## Results

### Anti-tumor effect of triptolide in various pancreatic cell lines

In vitro cell viability assay for triptolide in 6 PC cell lines (Capan-1, Capan-2, SNU-213, SNU-410, HPAFII, and Hs766T), the IC_50_ was 0.01 uM, 0.02 uM, 0.0096 uM for triptolide in Capan-1, Capan-2 and SNU-213. However, the growth of tumor cells was not significantly reduced by triptolide in Hs766T, SNU-410 and HPAFII (Fig. 1a).

To investigate distinction of gene expression in the sensitivity of triptolide, the gene expression analysis was performed in 6 PC cell lines (Capan-1, Capan-2, SNU-213, Hs766T, SNU-410 and HPAFII). The mRNA microarray data were constructed based on Pearson correlation distance and average linkage method. Five hundred twenty-nine genes were differently distributed on between PC cell lines with and without the sensitivity to triptolide (Fig. 1b).

**Fig. 2:**
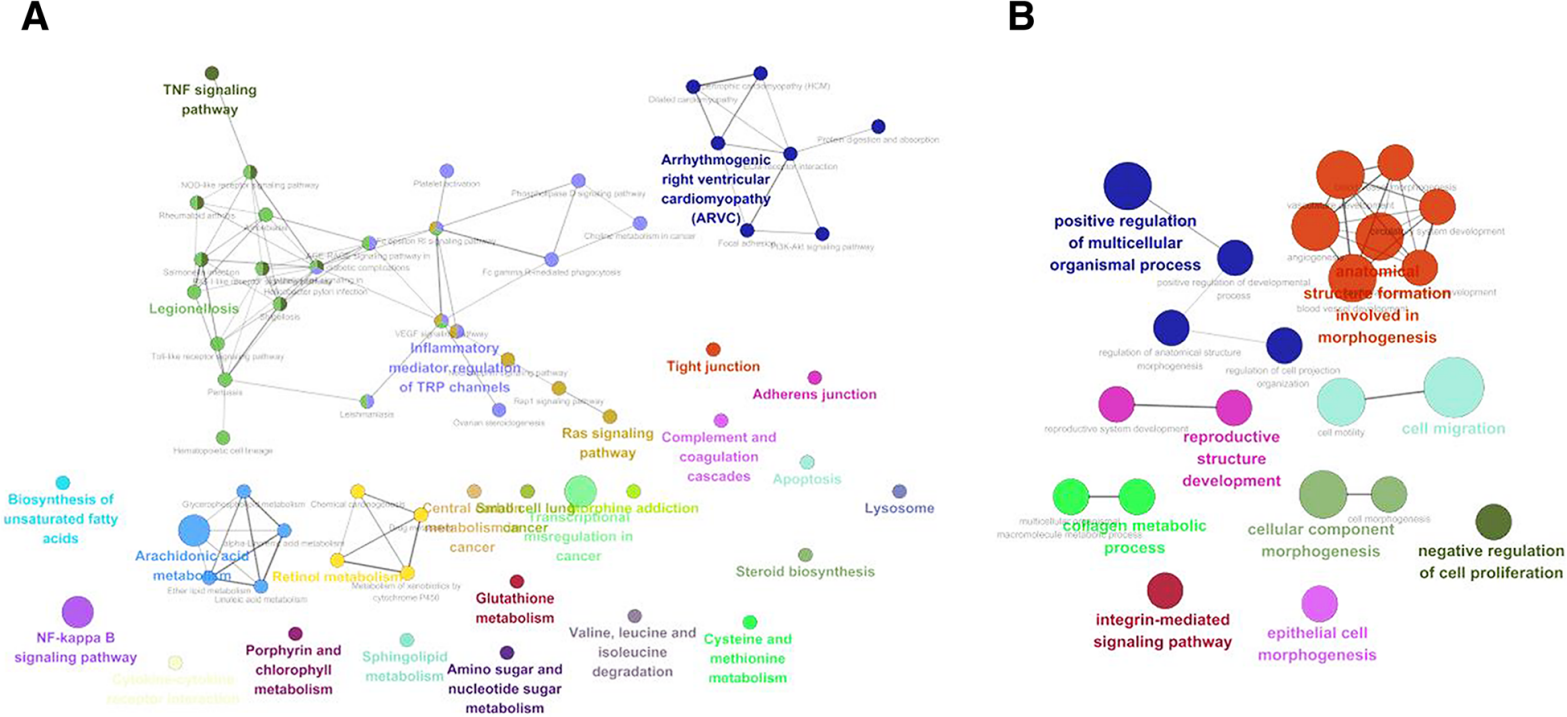
ClueGO network for top correlating DEGs. The size of the nodes reflects the statistical significance of the terms. A term can be included in more than one group. Different groups were colored differently. The group leading term (in bold) is the most significant term of the group. **a** A ClueGO network in KEGG for DEGs. **b** A ClueGO network in GO_BP for DEGs (*P*-value < 0.05)

To understand the biochemical, cellular, or biological functions in the large list of DEGs, we further analyzed a comprehensive functional gene ontology (GO) using KEGG and GO_BP tool. In analysis of pathway using gene expression profiling, the integrin mediated RAS signal pathway was associated with the sensitivity of the triptolide among PC cell lines (Fig. 2 and Additional file 1: Table S1).

### Cell viability assay with triptolide and immunoblot assay using pancreatic cancer (PC) patients derived cells (PDCs)

**Fig. 3:**
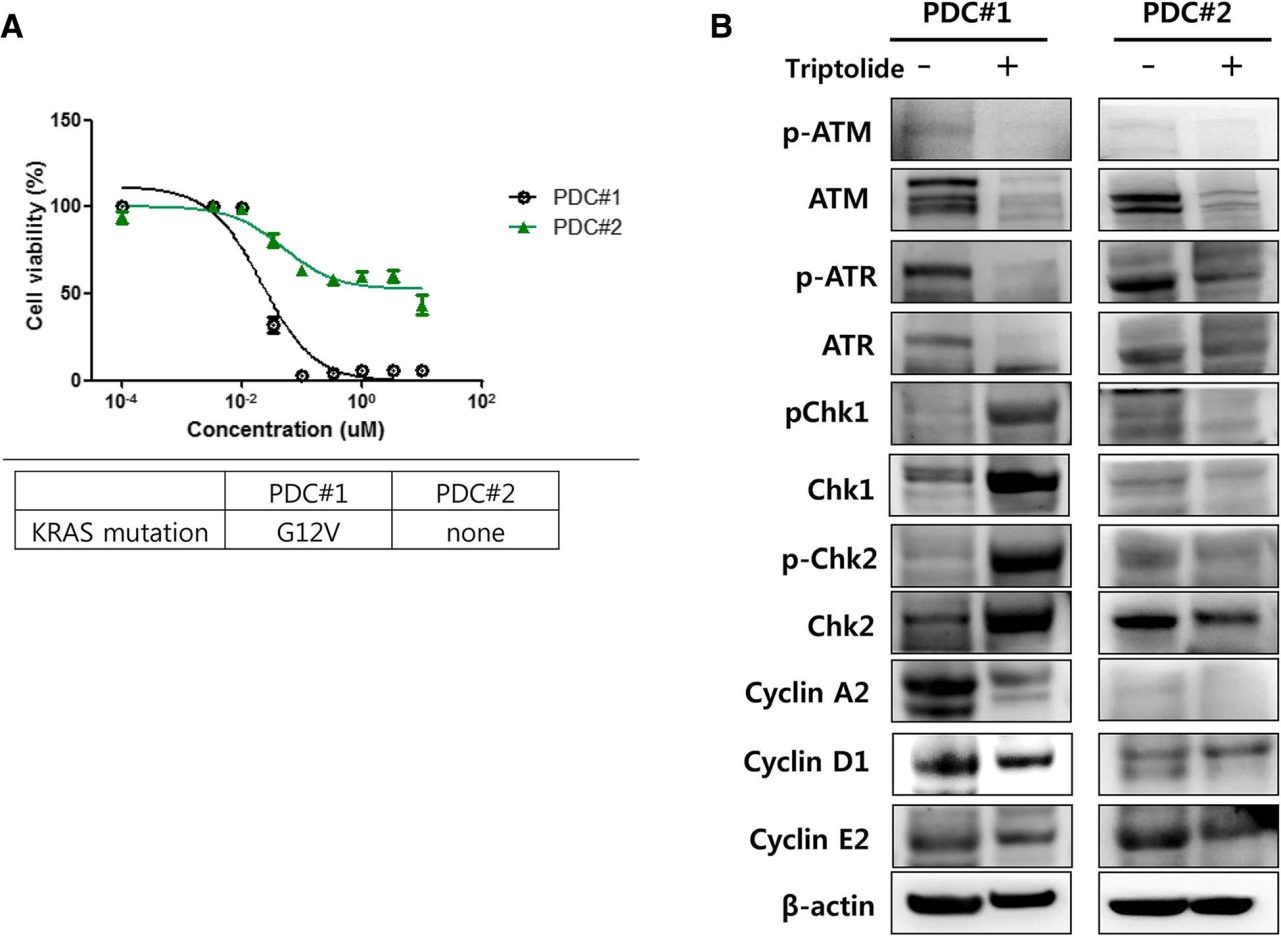
Effect of triptolide on PDCs. **a** Cell proliferation inhibition curve of triptolide on PDCs depend on KRAS mutation. **b** Immunoblot analysis of DNA damage response molecules

**Fig. 4:**
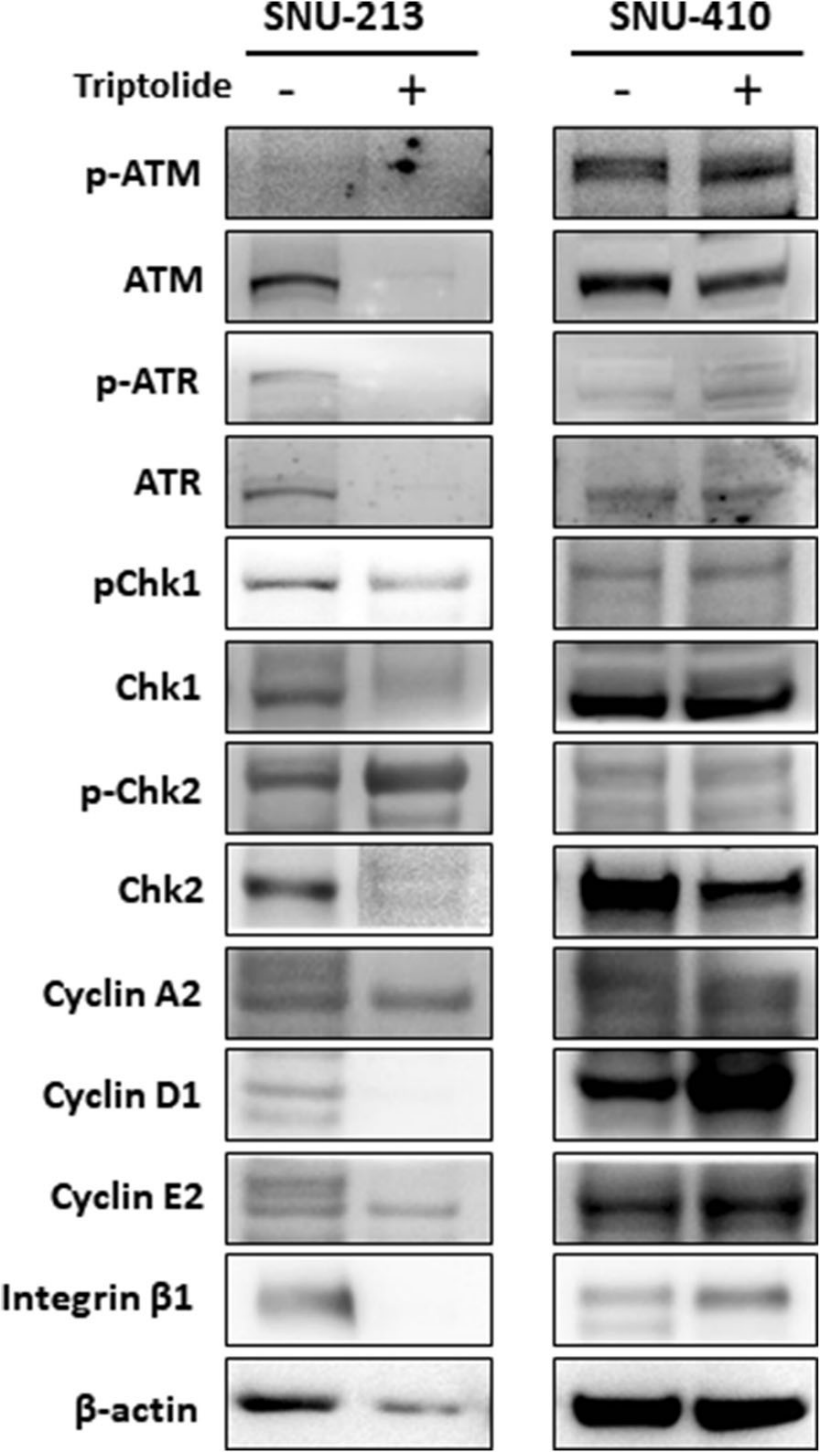
Effect of triptolide on DNA damage response in pancreatic cancer cell line: Immunoblot analysis of markers in DNA damage response

PDCs were established from metastatic lesions of KRAS G12 V mutant pancreatic cancer patient (PDC#1) and KRAS wild type patient (PDC#2), respectively, as described in the Material and methods section. We also confirmed the KRAS G12 V mutation in PDCs by ddPCR. First, we conducted the cell viability assay with triptolide using above two PDCs. Cell viability assays showed that triptolide suppressed the cell viability of only PDCs with KRAS G12 V mutation. PDCs with KRAS wild type was not inhibited by triptolide (Fig. 3a). We also analyzed the regulation of DNA damaged signals such as ATM/ATR/Chk1/Chk2 upon exposure to triptolide by immunoblot assay using PDCs. After triptolide, Chk2 phosphorylation was distinctively observed in PDCs sensitive to triptolide but, not PDCs insensitive to triptolide (Fig. 3b). This finding was consistent to that of immunoblot assay using cell lines (Fig. 4).

## Discussion

The treatment for metastatic pancreatic cancer need to be advanced. Rapid advances in molecular technologies together with improved algorithms for detecting specific molecular aberrations have made it feasible to perform biomarker-matched treatment [19, 20]. However, the role of molecular targeted therapy has not been established in pancreatic cancer. Although about 90% of pancreatic cancers harbor activated driver oncogenic KRAS, effective molecular targeted agent against KRAS mutation has not been developed until now [9]. Herein, we demonstrated that triptolide is a potent inhibitor in pancreatic cell lines and KRAS mutant PDCs. Consistent with the inhibition of cellular growth, we also observed the phosphorylation of Chk2 in cell line and PDCs sensitive to triptolide but, not in those insensitive to triptolide. The Chk2 phophorylation suggested that triptolide had the anti-tumor effect through inducing the apoptosis of tumor cells. These findings suggest that triptolide might be a promising targeted therapy for pancreatic cancer patients with KRAS mutant type.

Triptolide is a diterpene triepoxide which causes cell death in pancreatic, colon and breast cancer as well as cholangiocarcinoma and neuroblastoma cells among others [13, 21, 22]. Triptolide has been demonstrated to possess potent antitumor activity and induce apoptosis in a variety of tumor types in vivo and in vitro [12–14]. Triptolide has been reported to effect multiple pathways such as HSP70, HSF1, and Bcl-2 families [13, 23, 24]. In our pathway analysis using gene expression profiling, we could find that the antitumor effect of triptolide is associated with integrin mediated pathway. Integrins has been known to regulate the apoptotic response to DNA damage [25]. In this aspect, our outcome of analysis for pathway is well matched to the theoretical mechanism of triptolide. Additionally, in the immunoblot assay, we found that Chk2 phosphorylation was distinctively observed in PDCs sensitive to triptolide but, not PDCs insensitive to triptolide. Chk2 phosphorylation suggested that the antitumor effect to triptolide was associated with the apoptotic response [26, 27].

Our data was shown that genes of RAS pathway were differentially expressed between sensitive and non-sensitive cell lines and PDC with KRAS mutation was more sensitive to triptolide than without KRAS mutation. It has been reported that anti-tumor effect of triptolide is related to integrin-mediated RAS signaling pathway and DNA damage response in cancer [28]. However, further evaluation of the dependency of KRAS mutation to apoptosis via Chk2 phosphorylation is required to elucidate the mechanism.

KRAS mutation subtypes might induce different tumor biology in the same tumor type [29–33]. In pancreatic cancer and non-small cell lung cancer (NSCLC), the mutation subtype G12D and G12 V were associated with the different PI3K/AKT and MEK casade. In ovary serous carcinoma, patients with KRAS G12 V mutation had poor survival than those with KRAS G12D mutation or KRAS wild type [34]. Although triptolide showed remarkable antitumor activity in KRAS G12 V mutant PDCs in present study, our study dealt with only one PDC with KRAS mutation. Thus, our data needs to be interpreted with the caution. Our gene expression analysis based on pancreatic cancer cell lines also showed that different KRAS mutation subtypes had different gene expression profiling. These suggested that KRAS mutation subtypes might be robust biomarker to triptolide. Thus, which specific subtype of KRAS mutation is sensitive to triptolide needs to be resolved.

Clinical application of triptolide was limited because it was poorly soluble in water but soluble in organic solvent. To overcome problems with solubility, a highly water soluble analogue of triptolide, named minnelide has been designed and synthesized [11]. Recently, phase I clinical trial using minnelide in gastrointestinal tumor has been completed (NCT01927965). Currently, phase II clinical trial of minnelide in refractory pancreatic cancer is on going (NCT03117920). Our findings might be helpful to completely capture the subset of patients who may benefit to minnelide. More robust biomarkers and careful clinical trial design using minnelide are warranted.

## Conclusion

Our findings might be helpful to completely capture the subset of patients who may benefit to tripolide (minnelide). Triptolide showed antitumor activity in KRAS G12 V mutant PDCs in present study, further elucidation of KRAS mutation dependency of apoptosis via Chk2 phosphorylation is required. More robust biomarkers and careful clinical trial design using triptolide (minnelide) are warranted.

## Additional file

Additional file 1: **Table S1.** Top 20 genes upregulated in sensitive cell lines to triptolide. (XLSX 10 kb)

## Abbreviations

PC: Pancreatic cancer
PDC: Patient derived cell
